# Obituary

**Published:** 1850-01

**Authors:** 


*Obituary.*—Died at Pelham, Grundly Co. Tennessee, Dr Callender. A correspondent announcing the decease of Dr. Callender in the following extract from a private letter which we take the liberty of inserting.

"Dr. Callender, if I mistake not, was a reader of you Journal. He was one of the most eminent physicians of middle Tennessee. By his own uuaided efforts, he raised himself from the poor orphan boy, doing errands in the street, to the enviable position of being the most distinguished physican of his own, or any of the adjoining counties, He was a graduate of Transylvania University. He was devoted to his profession, regarding it as the highest calling to which a man could aspire. He was a stern opposer of empiricism in all its varied forms. He died at the age of about 30 years, with Typhoid Fever." J. C. S.

